# Coarse-Grained Drift Fields and Attractor-Basin Entropy in Kaprekar’s Routine

**DOI:** 10.3390/e28010092

**Published:** 2026-01-12

**Authors:** Christoph D. Dahl

**Affiliations:** Graduate Institute of Mind, Brain and Consciousness, Taipei Medical University, New Taipei City 235, Taiwan; christoph.dahl@tmu.edu.tw

**Keywords:** Kaprekar’s routine, finite dynamical systems, entropy funnels, basins of attraction, information-theoretic analysis, Markov coarse-graining, digit-gap features, gap-space dynamics

## Abstract

Kaprekar’s routine, i.e., sorting the digits of an integer in ascending and descending order and subtracting the two, defines a finite deterministic map on the state space of fixed-length digit strings. While its attractors (such as 495 for D=3 and 6174 for D=4) are classical, the global information-theoretic structure of the induced dynamics and its dependence on the digit length *D* have received little attention. Here an exhaustive analysis is carried out for D∈{3,4,5,6}. For each *D*, all states are enumerated and the transition structure is computed numerically; attractors and convergence distances are obtained, and the induced distribution over attractors across iterations is used to construct “entropy funnels”. Despite the combinatorial growth of the state space, average distances remain small and entropy decays rapidly before entering a slow tail. Permutation symmetry is then exploited by grouping states into digit multisets and, in a further reduction, into low-dimensional digit-gap features. On this gap space, a first-order Markov approximation is *empirically estimated* by counting one-step transitions induced by the exhaustively enumerated deterministic map. From the resulting empirical transition matrix, drift fields and the stationary distribution are computed *numerically*. These quantities serve as descriptive summaries of the projected dynamics and are not derived in closed form.

## 1. Introduction

Iterated digit transforms provide simple yet surprisingly rich examples of finite dynamical systems. Among them, Kaprekar’s routine occupies a special place: starting from a *D*-digit integer with at least two distinct digits, its digits are sorted into descending and ascending order, interpreted as integers, and subtracted. Formally, for a *D*-digit state *x* with digits $\left(d_{1},\ldots ,d_{D}\right)$, define

(1) $$\begin{array}{cc} \mathrm{desc}(x) & =\mathrm{integer}\;\mathrm{formed}\;\mathrm{by}\;\mathrm{sorting}\;\mathrm{the}\;\mathrm{digits}\;\mathrm{of}\;x\;\mathrm{in}\;\mathrm{descending}\;\mathrm{order},\\ \mathrm{asc}(x) & =\mathrm{integer}\;\mathrm{formed}\;\mathrm{by}\;\mathrm{sorting}\;\mathrm{the}\;\mathrm{digits}\;\mathrm{of}\;x\;\mathrm{in}\;\mathrm{ascending}\;\mathrm{order}. \end{array}$$

The Kaprekar map is then given by

(2) $$K_{D}\left(x\right)=\mathrm{desc}\left(x\right)-\mathrm{asc}\left(x\right),$$

iterated on the finite state space of *D*-digit integers (with leading zeros allowed). For D=4, this process famously converges to the attractor 6174 for almost all initial conditions, while 495 plays an analogous role for D=3 [1,2,3]. These facts are well known in recreational mathematics, and classical work has established existence, uniqueness, and basic properties of Kaprekar attractors in various bases and digit lengths [4,5,6,7,8,9]. Beyond these combinatorial results, however, relatively little is known about the *global* organisation of Kaprekar dynamics, its information-theoretic signatures, or how these properties depend on the digit length *D*. Seen as a finite dynamical system, Kaprekar’s routine raises a few concrete questions: (1) How are basins of attraction distributed as *D* increases, and how dominant is the largest basin? (2) How quickly does uncertainty about the eventual attractor collapse under iteration, when starting from a uniform prior over states? (3) Are there simple low-dimensional features of the digits that control whether a state is “easy” or “hard” to reach? (4) Can the dynamics be captured, at least approximately, by a stochastic process on a coarse-grained state space? Recent mathematical work has addressed structural and asymptotic properties of Kaprekar-type maps in a variety of settings, including *b*-adic generalisations, bounds on Kaprekar constants, and detailed analyses of two- and three-digit routines [10,11,12,13]. These studies highlight the richness of the underlying number-theoretic structure, but they do not aim to characterise the induced dynamics in information-theoretic or probabilistic terms. The present analysis focuses on base-10 Kaprekar maps with digit lengths D∈{3,4,5,6}. This range is small enough that the full state spaces can be enumerated exactly and all attractors and basins can be characterised without approximation, while still exhibiting clear changes in dynamical structure with increasing *D*. No asymptotic analysis in the limit of large digit length is attempted. Instead, the small-*D* regime is treated as a fully tractable model system on which information-theoretic and coarse-graining tools can be tested. Supplementary robustness checks in Appendix A.2 repeat selected analyses in base 8 to assess which qualitative conclusions are stable across bases.

Here a different angle is taken; instead of trying to obtain closed-form expressions for constants or loops, the Kaprekar map is treated as a finite directed graph, and questions are posed about its global statistics and coarse-grained descriptions: how entropy contracts, how permutation symmetries can be exploited, and how far simple ‘gap’ features go in explaining the dynamics. Standard tools from information theory and Markov-chain analysis [14,15,16] are used. In the present work these questions are addressed for D∈{3,4,5,6} by combining exhaustive enumeration with information-theoretic and statistical tools. The map *K* in (2) is treated as a deterministic update on a finite directed graph, and its structure is analysed at three complementary levels. First, at the level of individual states and attractors, all *D*-digit states with at least two distinct digits are enumerated, their attractors and distances (numbers of iterations) to convergence are obtained, and basin sizes are quantified. This representation makes it possible to construct *entropy funnels*: starting from a uniform distribution over states, the evolving distribution over attractors among the trajectories that have already converged is followed across iterations and the corresponding Shannon entropy is measured, providing a quantitative notion of uncertainty reduction under the dynamics. Second, the permutation symmetry of digit strings is exploited by grouping states into equivalence classes with identical digit multisets. This reduction yields a more compact representation that still respects the combinatorial constraints of the map. Multiset-level statistics are used to characterise how class sizes are distributed, how far typical classes lie from their attractors, and how different attractors are assembled from contributions of many small versus a few large classes. Third, a low-dimensional description is introduced in terms of simple digit-gap features. From the exact deterministic dynamics, empirical one-step transition frequencies between gap states can be estimated, yielding a first-order Markov approximation on this coarse-grained space. On this gap space, transition probabilities, stationary distributions, flow fields, and drift statistics are computed, and the resulting structure is related back to the basins and distances in the original state space. In addition, a simple linear regression framework is used to probe how far gap-based and aggregate digit features can account for the distance to attractor, viewed here as a notion of “difficulty” of reaching an attractor. All transition and stationary quantities reported for the gap-space chain are computed numerically from exhaustive enumeration; no closed-form derivation is claimed.

These three levels of description (states, multisets, and gap space) give a multiscale view of Kaprekar’s routine: from individual trajectories and basins to symmetry-reduced classes and finally to a very low-dimensional Markov approximation. The same approach should extend to other digit-based transforms and, more generally, to deterministic maps where one cares about coarse-grained information flow.

## 2. Materials and Methods

### 2.1. State Space, Attractors, and Distances

Fix a digit length D≥3 and work in base 10. Let ${𝒮}_{D}$ denote the set of *D*-digit states with at least two distinct digits, allowing leading zeros. Throughout, states are represented as length-*D* digit strings (in base 10) with *leading zeros allowed*. Equivalently, each integer is padded to *D* digits before applying the Kaprekar step (e.g., for D=4, 100 is treated as 0100). All digit sorting operations are performed on these *D* digits, including any zeros. This convention, commonly adopted in the Kaprekar literature, ensures that ${𝒮}_{D}$ is closed under the map $K_{D}$. For each *D* considered, restricting the initial ensemble to states without leading zeros does not change the attractor cycles reached or the entry times τ(x) for those states; it only changes the induced basin weights under a uniform prior, because the prior mass over ${𝒮}_{D}$ is altered. Write $x\in {𝒮}_{D}$ as a *D*-tuple of digits $\left(d_{1},\ldots ,d_{D}\right)$. The descending and ascending orderings desc(x) and asc(x) are defined as in (1), and the Kaprekar map is given by (2). When restricted to digit length *D* it is written as $K_{D}:{𝒮}_{D}\to {𝒮}_{D}$.

Because ${𝒮}_{D}$ is finite and $K_{D}$ is deterministic, every trajectory is eventually periodic: for each $x\in {𝒮}_{D}$ there exist integers μ≥0 and l≥1 such that $K_{D}^{t+l}\left(x\right)=K_{D}^{t}\left(x\right)$ for all t≥μ. Accordingly, an attractor is defined as a periodic orbit (cycle) of $K_{D}$; fixed points are the special case l=1.

**Lemma 1** (Eventual periodicity on a finite state space)**.** *Let f:S→S be a function on a finite set S. Then for any x∈S the sequence $x,f\left(x\right),f^{2}\left(x\right),\ldots$ is eventually periodic: there exist μ≥0 and l≥1 such that $f^{t+l}\left(x\right)=f^{t}\left(x\right)$ for all t≥μ.*

**Proof.**  Since *S* is finite, the sequence must repeat a value: there exist 0≤i<j with $f^{i}\left(x\right)=f^{j}\left(x\right)$. Let μ=i and l=j−i. Determinism implies that $f^{t+l}\left(x\right)=f^{t}\left(x\right)$ for all t≥μ.    □

A periodic orbit (cycle) $C\subset {𝒮}_{D}$ is an *attractor* if it is a directed cycle of $K_{D}$: there exist l≥1 and distinct states $a_{0},\ldots ,a_{l-1}\in {𝒮}_{D}$ such that $C=\{a_{0},\ldots ,a_{l-1}\}$ and $K_{D}\left(a_{i}\right)=a_{(i+1)\;\mathrm{mod}\;l}$ for all *i*. Fixed points are the special case l=1 (then C={a} with $K_{D}\left(a\right)=a$).

Given an attractor cycle *C*, its (forward) *basin* is

(3) $$\begin{array}{c} B(C) \end{array}:=\left\{x\in {𝒮}_{D}:\begin{array}{c} K_{D}^{t}\left(x\right)\in C \end{array}\;\mathrm{for}\;\mathrm{some}\;t\geq 0\right\}.$$

For x∈B(C) the *distance to attractor* (cycle entry time) is defined as

(4) $$\mathrm{dist}\left(x\right):=\min\left\{t\geq 0:\begin{array}{c} K_{D}^{t}\left(x\right)\in C \end{array}\right\}.$$

It is convenient to write α(x) for the attractor cycle reached from *x* and τ(x):=dist(x) for the corresponding convergence time. The observed cycle structure for each D∈{3,4,5,6} (including whether l>1 occurs) is reported in Section 2.2 and Appendix A.3. For each D∈{3,4,5,6} the set ${𝒮}_{D}$ is enumerated, all attractor cycles are identified, basin sizes |B(C)| are computed, and dist(x) is recorded for all states.

### 2.2. Attractor Detection and Cycle Structure

For each digit length *D*, the map $K_{D}$ was iterated from every state $x\in {𝒮}_{D}$ until the trajectory first revisited a previously visited state. From this first repeat, the resulting periodic orbit (cycle) C(x) and its length l(x)=|C(x)| were extracted and recorded. For D=3 and D=4, all attracting cycles had length one (fixed points). For D=5, all attractors were genuine cycles with l>1 (one 2-cycle and two 4-cycles). For D=6, both fixed points (l=1) and a dominant 7-cycle (l=7) were observed. The full cycle lists, lengths, and basin weights are provided in Appendix A.3.

### 2.3. Entropy Funnels

To quantify information funnels an initial distribution that is uniform over all non-trivial states for a given *D* is considered. For each iteration t≥0 define the subset of states that have already reached an attractor by time *t*,

(5) $$A_{t}:=\left\{x\in {𝒮}_{D}:\tau \left(x\right)\leq t\right\},\;N_{t}:=\left|A_{t}\right|.$$

Among these converged states, the empirical distribution over attractors at time *t* is

(6) $$p_{t}\left(a\right)=\frac{1}{N_{t}}\left|\{x\in A_{t}:\alpha \left(x\right)=a\}\right|,$$

where the sum over *a* runs over all attractors for the given *D*. The Shannon entropy

(7) $$H_{t}=-\sum_{a}p_{t}\left(a\right)\log_{2}p_{t}\left(a\right)$$

is then computed as a function of iteration *t*. For small *t* the distribution $p_{t}$ is dominated by attractors that are reached quickly; as *t* increases and more trajectories converge, $p_{t}$ approaches the basin size distribution. Plotting $H_{t}$ yields a raw entropy funnel. To compare decay profiles across *D*, the normalised entropy is plotted,

(8) $${\tilde{H}}_{t}=\frac{H_{t}-H_{\infty }}{H_{0}-H_{\infty }},$$

where $H_{\infty }$ denotes the basin size entropy, i.e., the entropy of the attractor distribution obtained once all states have converged ($t\geq t_{\max}:=\max_{x\in {𝒮}_{D}}\tau \left(x\right)$, so that $A_{t}={𝒮}_{D}$ and $p_{t}=p_{\infty }$), and in practice $H_{\infty }$ is set to $H_{T}$ with $T\geq t_{\max}$ in the computations. Thus ${\tilde{H}}_{0}=1$ and ${\tilde{H}}_{t}\to 0$ as t→∞.

### 2.4. Multiset Representation

Because permuting the digits of *x* does not affect the outcome of a Kaprekar step, many states form equivalence classes with identical digit multisets. Formally, an equivalence relation is defined on ${𝒮}_{D}$ by x∼y if their digits coincide as multisets. The equivalence classes are in bijection with digit multisets and are referred to as *multiset classes*. Each class has a *size* (number of distinct permutations) and a well-defined mean distance to attractor obtained by averaging dist(x) over the states in the class. For each *D* all digit multisets, their class sizes, and their mean distances are enumerated. It is also recorded, for each attractor, how many states in its basin arise from each multiset class.

### 2.5. Gap Features and Markov Chain in Gap Space

To obtain a low-dimensional, permutation-invariant description of digit structure, two simple “gap” features are used (defined formally in Equation (9)). The first captures overall digit spread, while the second captures an internal separation that distinguishes configurations in which one or two extreme digits are separated from a more homogeneous bulk. In preliminary exploratory work, additional quantities such as further internal gaps, digit sum, and digit variance were inspected. Digit sum and digit variance are later included as predictors in the regression analysis, but $\left(g_{1},g_{2}\right)$ are adopted for the Markov-chain description because they already generate a compact and interpretable gap space. Appendix A.2 further compares induced occupancy and mean drift fields under alternative definitions of $g_{2}$, quantifying agreement via cellwise correlations and the mean cosine similarity of drift vectors.

For each state $x\in {𝒮}_{D}$ simple digit features are computed. For notational convenience, let $d_{\left(1\right)}\left(x\right)\geq d_{\left(2\right)}\left(x\right)\geq \ldots \geq d_{\left(D\right)}\left(x\right)$ denote the digits of *x* sorted in non-increasing order (including leading zeros). The gap features used for the gap-space projection are defined as

(9) $$g_{1}\left(x\right)=d_{\left(1\right)}\left(x\right)-d_{\left(D\right)}\left(x\right),\;g_{2}\left(x\right)=d_{\left(2\right)}\left(x\right)-d_{\left(3\right)}\left(x\right).$$

Here $d_{\left(k\right)}\left(x\right)$ denotes the *k*-th largest digit of *x*. The choice $g_{2}=d_{\left(2\right)}\left(x\right)-d_{\left(3\right)}\left(x\right)$ is intended to capture an *internal break* between the leading digits and the remaining digits. In particular, many digit configurations share the same overall spread $g_{1}$ but differ in whether there are two extreme digits separated from a more homogeneous bulk; this distinction is directly reflected in $d_{\left(2\right)}-d_{\left(3\right)}$. By contrast, the alternative $d_{\left(1\right)}-d_{\left(2\right)}$ primarily detects a single extreme digit and is therefore often partly redundant with $g_{1}$. To assess the robustness of the coarse-grained description, Appendix A.2 compares the induced flow fields obtained from several alternative definitions of $g_{2}$ and reports which qualitative conclusions are stable across choices.

These define a discrete set

(10) $$G_{D}:=\left\{\left(g_{1}\left(x\right),g_{2}\left(x\right)\right):x\in {𝒮}_{D}\right\}$$

of possible gap pairs $\left(g_{1},g_{2}\right)$.

Because the projection $x\mapsto g\left(x\right)=(g_{1}\left(x\right),g_{2}\left(x\right))$ is many-to-one, the induced dynamics on $G_{D}$ is generally non-deterministic: distinct digit strings sharing the same gap state *g* can transition to different successor gap states under the deterministic Kaprekar map $K_{D}$. An empirical first-order Markov approximation on this discrete grid is therefore defined by counting one-step transitions across the exhaustively enumerated map.

For each gap state $g=\left(g_{1},g_{2}\right)\in G_{D}$, consider the collection of underlying states

(11) $${𝒮}_{D}\left(g\right):=\left\{x\in {𝒮}_{D}:\left(g_{1}\left(x\right),g_{2}\left(x\right)\right)=g\right\}.$$

One Kaprekar step is applied to each $x\in {𝒮}_{D}\left(g\right)$, the successor gap state $g^{\prime }\left(x\right):=\left(g_{1}\left(K_{D}\left(x\right)\right),\;g_{2}\left(K_{D}\left(x\right)\right)\right)$ is recorded, and empirical transition frequencies

(12) $$P_{D}\left(g\to h\right):=\frac{1}{|{𝒮}_{D}\left(g\right)|}\left|\{x\in {𝒮}_{D}\left(g\right):g^{\prime }\left(x\right)=h\}\right|$$

are estimated.

Equivalently, letting $N_{D}\left(g,h\right)=\left|\{x\in {𝒮}_{D}:g\left(x\right)=g,\;g\left(K_{D}\left(x\right)\right)=h\}\right|$, one has $P_{D}\left(g\to h\right)=N_{D}\left(g,h\right)/\sum_{u\in G_{D}}N_{D}\left(g,u\right)$. The matrix $P_{D}$ is row-stochastic and summarises the average one-step behaviour of the full deterministic map after projection to the gap space.

A stationary distribution of the Markov approximation is any distribution $\pi_{D}$ on $G_{D}$ satisfying $\pi_{D}=\pi_{D}P_{D}$. In practice, the stationary distribution reported is obtained numerically by iterating $q_{t+1}=q_{t}P_{D}$ until convergence from the initial gap distribution induced by the uniform prior on ${𝒮}_{D}$; when the chain is ergodic, this is equivalent to computing the normalised left eigenvector of $P_{D}$ associated with eigenvalue 1.

For visualisation of gap-space flow fields, a drift vector is associated with each occupied gap state $g\in G_{D}$. Writing $g\left(x\right):=(g_{1}\left(x\right),g_{2}\left(x\right))$ and $\Delta g\left(x\right):=g\;\left(K_{D}\left(x\right)\right)-g\left(x\right)$, the empirical mean one-step drift at *g* is

(13) $$v_{D}\left(g\right):=\frac{1}{|{𝒮}_{D}\left(g\right)|}\sum_{x\in {𝒮}_{D}\left(g\right)}\Delta g\left(x\right).$$

Because ${𝒮}_{D}$ is exhaustively enumerated for each D∈{3,4,5,6}, this average includes *all* states *x* consistent with the gap pair *g*. To quantify within-cell heterogeneity (i.e., how much Δg(x) varies across different states *x* sharing the same gap pair *g*), the empirical covariance of increments is also computed,

(14) $$\Sigma_{D}\left(g\right):=\frac{1}{|{𝒮}_{D}\left(g\right)|-1}\sum_{x\in {𝒮}_{D}\left(g\right)}\left(\Delta g\left(x\right)-v_{D}\left(g\right)\right){\left(\Delta g\left(x\right)-v_{D}\left(g\right)\right)}^{⊤}.$$

Appendix A.1 reports representative examples and summary dispersion statistics, providing a direct visualisation of variability around the mean drift arrows.

These empirical probabilities define a first-order Markov chain on $G_{D}$ with transition matrix $P_{D}$, which provides a coarse-grained approximation to the projected dynamics on the gap space (the true projected process need not be strictly Markov). It should be emphasised that $P_{D}$ is used here as a descriptive first-order approximation to the projected dynamics on the gap space. Quantifying the approximation error (e.g., by comparing against higher-order models) is an important direction but is beyond the scope of the present work. The chain is therefore treated primarily as a compact summary of average flow patterns rather than an exact probabilistic model of the projected dynamics. The following quantities are computed: (1) the stationary distribution $\pi_{D}$ satisfying $\pi_{D}P_{D}=\pi_{D}$(computed numerically, e.g., as the normalised left eigenvector of $P_{D}$ or by power iteration); (2) the empirical distribution of gap states under the uniform prior on ${𝒮}_{D}$; and (3) average changes $\Delta g_{1}$ and $\Delta g_{2}$ per step for each gap state.

### 2.6. Predicting Distance to Attractor from Digit Features

To link local digit structure to distance to attractor, a simple regression analysis is performed. For each *D*, up to *N* = 50,000 states (or all states when fewer are available) are drawn uniformly without replacement from ${𝒮}_{D}$. For D=6 this corresponds to a random sample of approximately 5% of all admissible states, which keeps computation manageable while still covering a wide range of digit configurations. For each sampled state, $g_{1}$ and $g_{2}$ as defined above, the mean digit $\bar{d}\left(x\right)=D^{-1}\sum_{i=1}^{D}d_{i}\left(x\right)$, and the variance of digits $\mathrm{Var}\left(d(x)\right)$ are computed. All reported goodness-of-fit metrics are computed on held-out test data: for each *D*, the sampled states were split into 80% training and 20% test sets, the model in (15) was fit on the training set, and $R^{2}$ and RMSE were evaluated on the test set. All features are standardised (zero mean and unit variance). The mean digit $\bar{d}\left(x\right)$ is used instead of the raw digit sum to remove trivial scaling with *D*. A linear model

(15) $$\hat{\tau }\left(x\right)=\beta_{0}+\beta_{1}\;g_{1}\left(x\right)+\beta_{2}\;g_{2}\left(x\right)+\beta_{3}\;\bar{d}\left(x\right)+\beta_{4}\;\mathrm{Var}\left(d(x)\right)$$

is then fit by least squares, and, for each *D*, the coefficient of determination $R^{2}$, the root mean squared error (RMSE), and the learned weights β are reported. For the comparisons between “easy” and “hard” states in Figure 1, states are ranked by τ(x) and the fastest and slowest deciles (smallest and largest 10% of distances) are selected. Digit features are then averaged separately for these two groups. Throughout this subsection, linear regression is used deliberately as a simple and interpretable baseline. The model links distance to attractor to low-dimensional digit summaries in a way that allows direct inspection of feature weights and effect directions, at the cost of restricting attention to linear relationships. More flexible nonlinear models (e.g., decision trees or kernel-based methods) could in principle capture additional structure in the data but would make it harder to relate predictive performance back to specific digit features. For this reason, the present analysis treats the linear model as a reference point rather than an attempt to optimise predictive accuracy.

### 2.7. Numerical Setup

All computations were performed in MATLAB (R2024b, Mathworks^®^, Natick, MA, USA), using integer-valued operations for the Kaprekar map that are exactly represented in double-precision arithmetic for the ranges of *D* considered and double-precision arithmetic for derived quantities such as entropies. Enumeration of ${𝒮}_{D}$ and construction of the gap-space Markov chain are exact for each D∈{3,4,5,6}. Where sampling is used (in the regression analysis), the corresponding sample size is stated.

## 3. Results

### 3.1. Visualising Attractors and Basins

Before scalar summary statistics are introduced, the coarse-grained flow of Kaprekar dynamics in the gap space is visualised. Figure 2 shows, for each digit length D∈{3,4,5,6}, the occupancy of gap states $\left(g_{1},g_{2}\right)$ under a uniform prior over ${𝒮}_{D}$ together with the corresponding average one-step drift vectors.

Colours encode how many states realise a given gap pair, and arrows indicate the mean change $\left(\Delta g_{2},\Delta g_{1}\right)$ produced by a single Kaprekar step. For three digits, trajectories concentrate along a narrow band and converge towards a single high-occupancy region. For larger *D* the occupied region of the gap space broadens and the flow becomes more heterogeneous, with weaker and more dispersed drift, foreshadowing the more fragmented basin structure quantified below.

Because each gap cell $g=(g_{1},g_{2})$ aggregates many distinct digit configurations, the arrows in Figure 2 represent *conditional means* of the increment Δg(x) over all states consistent with that cell. Table 1 quantifies how much increment directions vary *within* a fixed gap cell across digit lengths, using the circular (angular) dispersion $\sigma_{\theta }\left(g\right)$. Directional variability is numerically negligible for D=3,4 but becomes non-negligible for D≥5. A representative D=6 example is visualised in Appendix A.1. This within-cell measure makes the qualitative notion of “more dispersed drift” in Figure 2 explicit.

Figure 3 summarises how the global structure of Kaprekar dynamics changes with the number of digits *D*. For each *D* the following summary quantities are reported: the number of distinct attractors, the fraction of states lying in the largest basin of attraction, the mean and median distance to attractor, and the maximal distance observed. Here the distance to attractor measures how many iterations of the Kaprekar map are needed before the trajectory enters its attractor cycle. Despite the combinatorial explosion of the state space as *D* increases, the average number of iterations needed to reach an attractor remains small for all values of *D* considered. By contrast, the maximal distance, the dominance of the largest basin, and the number of distinct attractors all vary systematically with *D*. For D=3 and D=4, a single attractor dominates the dynamics, in the sense that most initial states eventually flow into one large basin. For D=5 and D=6, the picture is more fragmented: the largest basin occupies a much smaller fraction of the state space and many additional, smaller attractors appear.

For D∈{5,6} the long-run attractor distribution under the uniform initial ensemble does not collapse to a single attractor: instead, multiple attractor cycles (including fixed points as the special case l=1) carry non-zero basin weight. Consequently, the limiting attractor entropy $H_{\infty }=H\left(p_{\infty }\right)$ is strictly positive, where $p_{\infty }$ denotes the basin size distribution over attractor cycles (i.e., $p_{\infty }\left(C\right)=|\left\{x\in {𝒮}_{D}:\alpha \left(x\right)=C\right\}\left|/\right|{𝒮}_{D}|$). Table 2 summarises the number of distinct attractor cycles and the dominant basin weights for D=5,6, with full attractor lists (including cycle lengths) and basin sizes provided in Appendix A.3. Representative numerical examples of convergence to distinct attractor cycles are reported in Appendix A.3.

### 3.2. Entropy Funnels Across Digit Lengths

Information contraction under Kaprekar iteration can be summarised by *entropy funnels*, which track how uncertainty about the eventual attractor decreases over time. For each digit length *D*, Figure 4 shows the Shannon entropy of the induced distribution over attractors, among the trajectories that have converged by iteration *t*, as a function of iteration, together with a normalised version. High entropy corresponds to a situation in which many attractors are still plausible, whereas low entropy indicates that almost all initial states have effectively committed to a small subset of attractors. Raw entropy (in bits) decays rapidly in the first few iterations and then flattens as trajectories approach their attractors and the basin size distribution is revealed. This apparent “incomplete” decay is expected under the chosen conditioning: $H_{t}$ is computed on the subset of trajectories that have converged by iteration *t*, so the composition of the conditioned set changes with *t* as progressively slower trajectories enter it. Consequently, late-time changes in $H_{t}$ primarily reflect the basin weight distribution revealed by these late arrivals (a compositional effect), rather than continued uncertainty within trajectories already included in the conditioned set. The normalised curves show that, for all *D*, the bulk of uncertainty about the eventual attractor is resolved within roughly five iterations, but the final residual entropy depends strongly on the number and relative sizes of basins. For D=3 and D=4, the normalised entropy drops close to zero, reflecting the near-complete dominance of a single attractor. For D=5 and D=6, the decay is less complete, consistent with the proliferation of attractors and a more even distribution of basin sizes, so that some uncertainty about the final attractor remains even after many iterations.

### 3.3. Multiset Structure and Digit-Level Features

Factoring states into digit multisets provides a compact summary of the combinatorial structure of the map (Figure 5). A digit multiset records which digits appear and how often but ignores their order, so all permutations of the same digits belong to the same multiset class. This perspective separates purely combinatorial effects (how many permutations a given multiset admits) from dynamical effects (how quickly states built from that multiset converge). For each digit length D∈{3,4,5,6}, the left-hand panel shows the distribution of multiset sizes, i.e., the number of distinct permutations in each class, both as absolute counts (solid line, left axis) and as normalised probabilities (dashed line, right axis). As *D* increases, the number of multiset classes grows and the distributions shift towards larger sizes with heavier upper tails, indicating the appearance of rare digit patterns with many distinct permutations. The right-hand panels display the corresponding distributions of mean distance to attractor per multiset, obtained by averaging dist(x) over all states in a class. For all *D*, most multisets are associated with short mean distances, but the tails extend further for D=5 and D=6, reflecting a growing minority of digit patterns that are systematically linked to longer transients. This multiset-level view shows that part of the complexity at higher digit lengths arises from an increasingly uneven allocation of basin structure across combinatorial classes.

For each digit length *D*, states are ranked by their distance to attractor and divided into two extreme groups: the fastest 10% are referred to as “easy” states and the slowest 10% as “hard” states. Comparing their digit features reveals systematic differences (Figure 1). Easy states tend to have a larger overall digit spread $g_{1}=\max\left(d\right)-\min\left(d\right)$, and for some *D* they also show higher digit variance, indicating that configurations whose digits are more widely dispersed are typically closer to an attractor in the dynamical sense. Differences in digit sum are much less informative: because the total sum of digits naturally increases with *D*, shifts in this feature are largely driven by digit length rather than by dynamical difficulty and should therefore be interpreted with caution.

### 3.4. Gap-Space Markov Structure and Drift

In the gap space, the projection onto $\left(g_{1},g_{2}\right)$ yields an empirical first-order Markov approximation (Section 2.5) whose flow fields are summarised in Figure 2. For D=3, the occupied region of the gap space forms a narrow wedge constrained by $g_{2}\leq g_{1}$, with the highest occupancy at moderate $g_{1}$ and small-to-moderate $g_{2}$. The corresponding drift vectors show a coherent flow towards larger $g_{1}$ and moderate $g_{2}$, foreshadowing the basin structure quantified below. For larger *D*, the flow patterns become progressively more asymmetric, and the stationary distributions place most of their mass in regions with large $g_{1}$ and moderate $g_{2}$, indicating a bias towards states with a wide overall digit spread but only a moderate gap between the second and third largest digits. Average drift vectors show a robust tendency to increase $g_{1}$ and decrease $g_{2}$, with drift magnitudes decreasing as *D* grows. To summarise these trends across digit lengths, one-step changes $\Delta g_{1}$ are regressed on $g_{2}$ and $\Delta g_{2}$ on $g_{1}$ for each *D*, and mean changes per step are computed (Figure 6). The slopes of the linear relations $\Delta g_{1}\approx a_{D}g_{2}+b_{D}$ and $\Delta g_{2}\approx c_{D}g_{1}+d_{D}$ are negative for all *D*, but their magnitude decreases with *D*, indicating that the coupling between the two gap coordinates weakens in higher-digit systems. From a more intuitive perspective, the dynamics tend to push states towards regions of the gap space where one large overall digit range coexists with a more balanced configuration among the middle digits, but this directional bias becomes less pronounced as *D* grows.

### 3.5. Predictability of Distance from Digit Features

The regression analysis (Figure 7) quantifies how well this particular choice of simple digit features predicts distance to attractor. For D=3, the linear model explains approximately $R^{2}\approx 0.43$ of the variance in distance, with a root mean squared error (RMSE) of about 1.08 steps over N=998 states. For D=4,5,6, the $R^{2}$ values drop to roughly 0.02–0.03 with a markedly larger RMSE, indicating that, beyond three digits, only a very small fraction of the variability in distance to attractor can be accounted for by these four standardised features within a linear model. In other words, for D≥4, the link between this particular low-dimensional linear summary of local digit structure and global convergence time is weak. The learned regression weights highlight how the influence of individual features changes with *D*. For D=3, a large digit spread $g_{1}$ is strongly associated with faster convergence, and digit variance carries a clear positive weight, suggesting that states with more heterogeneous digits tend to approach their attractors quickly. For larger *D*, the weights shrink in magnitude and fluctuate in sign across features, consistent with a more entangled and higher-dimensional dependence of distance to attractor on the underlying digit configuration. Together, these results show that in the three-digit case simple low-dimensional linear descriptors based on $\left(g_{1},g_{2},\mathrm{digit}\;\mathrm{sum},\mathrm{digit}\;\mathrm{variance}\right)$ provide a substantially informative proxy for dynamical difficulty, whereas for D≥4 these particular features, within a linear model, account for only a small fraction of the variability in distance to attractor. Richer feature sets or nonlinear models might recover additional structure but are beyond the scope of the present analysis.

Standard regression diagnostics were examined for all models. Residuals showed no systematic dependence on fitted values, and Q–Q plots indicated moderate deviations from normality, particularly for larger *D*, reflecting the discrete and bounded nature of the response variable. Given the large sample sizes, inference on regression coefficients is robust, and the conclusions rely on effect size ($R^{2}$ and RMSE) rather than strict distributional assumptions.

## 4. Discussion

The results above describe Kaprekar’s routine, for D=3–6, as a finite dynamical system seen through several information-theoretic lenses. A very simple digit transform already produces a surprisingly layered structure: shallow typical transients but long tails, a shift from one dominant basin to many smaller ones, and a useful, but ultimately limited, low-dimensional gap description. At the coarsest level, the global summaries in Figure 3 indicate that the Kaprekar dynamics remains shallow on average across all digit lengths considered: typical trajectories reach an attractor in only a few iterations, despite the combinatorial growth of the state space. This shallow behaviour coexists with marked changes in extremal and structural quantities. The maximal distance to attractor increases with *D*, the dominance of the largest basin decreases, and the number of distinct attractors rises. For three and four digits, the dynamics are effectively governed by a single large basin, whereas for five and six digits the state space splits into many smaller basins with more heterogeneous sizes. This transition from “one big funnel” to a more fragmented landscape is a first indication that, within the range D=3–6 examined here, the global organisation of the map shifts from dominance by a single large basin to a heterogeneous collection of smaller basins. The entropy funnels in Figure 4 provide a complementary information-theoretic view. Starting from a uniform prior over states, the induced distribution over attractors among the trajectories that have converged exhibits a rapid initial entropy drop, followed by a slower tail. In more concrete terms, most uncertainty about the eventual attractor is resolved within a handful of iterations, but a small residual uncertainty can persist for many steps, especially when many attractors of comparable basin size are present. For D=3 and D=4, the normalised entropy curves fall close to zero, in line with the near-monopolisation of the state space by a single attractor. For D=5 and D=6, the residual entropy remains appreciable, reflecting the proliferation of attractors and a more even spread of basin sizes. Thus, entropy funnels capture, in a single scalar time series, both the fast collapse of uncertainty and the dependence of long-term behaviour on basin geometry.

Factoring states into digit multisets yields a first level of symmetry reduction that separates combinatorial and dynamical effects (Figure 5). On the combinatorial side, both the number of multiset classes and their typical sizes grow quickly with *D*, and the size distributions develop heavier upper tails. This indicates the appearance of rare digit patterns that admit many distinct permutations. On the dynamical side, the distributions of mean distance to attractor per multiset remain concentrated on short distances but develop longer tails for D=5 and D=6. A small subset of multisets is therefore systematically associated with longer transients. In other words, as *D* increases the basin structure is distributed more unevenly across multisets: a few digit patterns dominate large basins, while many others sit in small, long-tailed classes. The analysis of “easy” and “hard” states in Figure 1 moves one step closer to the digit level. By contrasting the fastest and slowest deciles of distance to attractor, simple patterns emerge: easy states tend to have a larger overall digit spread $g_{1}$ and, for some digit lengths, higher digit variance. Configurations whose digits are widely dispersed therefore tend, on average, to reach an attractor more quickly. In contrast, the total digit sum carries much less interpretable dynamical information once trivial scaling with *D* is taken into account. These observations suggest that certain aspects of “difficulty” are already visible in low-level digit statistics, even before more refined features are introduced.

Gap space provides a coarser, but highly structured, view of the dynamics. By tracking only the overall spread $g_{1}$ and the internal gap $g_{2}$ between the second and third largest digits, Kaprekar’s routine induces an empirical first-order Markov chain on a relatively small grid. The resulting flow fields and drift statistics reveal a consistent directional bias: on average, one Kaprekar step tends to increase $g_{1}$ and decrease $g_{2}$, pushing states towards regions with a wide overall digit range but a more balanced configuration among the middle digits (Figure 6). For three digits, successor states concentrate near the diagonal $g_{1}\approx g_{2}$, and the coupling between $g_{1}$ and $g_{2}$ is strong. As *D* increases, the stationary distributions shift and the linear relations between $\Delta g_{1}$ and $g_{2}$, and between $\Delta g_{2}$ and $g_{1}$, weaken in slope. This weakening suggests that, in higher-digit systems, the two gap coordinates no longer suffice to capture the dominant directions of flow and that additional degrees of freedom in the digit space become dynamically relevant. The gap-space Markov chain is therefore best viewed as a first-order coarse-grained description: it captures the main flow patterns in $\left(g_{1},g_{2}\right)$ but is not intended as an exact probabilistic model of the projected dynamics. The regression analysis in Figure 7 makes this limitation explicit. For D=3, a simple linear model based on $\left(g_{1},g_{2},\mathrm{digit}\;\mathrm{sum},\mathrm{digit}\;\mathrm{variance}\right)$ explains a substantial fraction of the variance in distance to attractor, with errors on the order of one step. In this regime, low-dimensional digit summaries provide a reasonably informative proxy for dynamical difficulty. For D=4,5,6, however, the coefficient of determination $R^{2}$ drops to a few percent and the learned feature weights shrink and fluctuate in sign. Beyond three digits, the relationship between local digit structure and global convergence time becomes increasingly high-dimensional and entangled, so that linear combinations of a small number of simple features capture only a small part of the behaviour. This breakdown of low-dimensional predictability within the present feature set is consistent with the more fragmented basin geometry and more heterogeneous multiset patterns observed at larger *D*.

Several limitations and extensions follow naturally from these findings. The present study is numerical and restricted to base 10 and digit lengths up to six. From a mathematical perspective, one natural direction is to seek analytic bounds on entropy decay, basin sizes, or gap-space drift as *D* grows, perhaps by exploiting known structural results on Kaprekar constants and loops in higher bases. Another direction is to refine the coarse-graining schemes: alternative feature sets, nonlinear mappings into the gap space, or higher-order Markov projections could be explored to recover part of the predictive power lost at larger *D*. The regression framework could also be extended beyond linear models, for example by investigating whether low-depth decision trees or other simple classifiers find more informative digit combinations without sacrificing interpretability.

More broadly, the picture that emerges is that of a finite information-processing system that rapidly funnels a large set of inputs into a much smaller set of outputs while retaining enough internal structure to defeat simple low-dimensional representations of the dynamics once the state space becomes sufficiently large. Kaprekar dynamics therefore offer a useful model system for studying information funnels and coarse-grained Markov structure in deterministic maps on finite spaces. Similar patterns occur in other settings: in statistical physics, many microscopic configurations are summarised by a few macroscopic variables, such as temperature or magnetisation [17,18]; in machine learning, deep networks and information bottleneck methods restrict information flow through low-dimensional latent or bottleneck layers, yielding compact internal codes that still support accurate predictions [19,20]; and in decision neuroscience, multiple streams of evidence are modelled as being accumulated into a single decision variable that governs choice [21,22,23]. Against this background, the Kaprekar map provides a fully tractable model system: the entire state space and all attractors are known explicitly, yet the induced information funnels and coarse-grained dynamics remain non-trivial. This makes the Kaprekar map a natural model system for testing methods that search for informative coarse-grainings or low-dimensional summaries and for exploring how such techniques might carry over to more biologically motivated dynamical models.

## Figures and Tables

**Figure 1:**
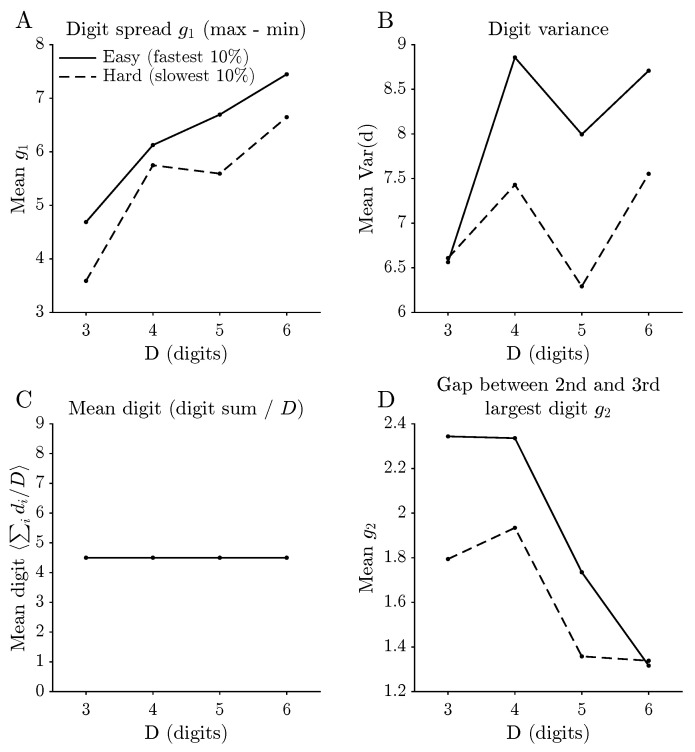
Digit features for easy and hard Kaprekar states. For each digit length *D*, states are split into the fastest and slowest deciles of distance to attractor. Panels (**A**–**D**) show, respectively, the mean digit spread $g_{1}$ (difference between the largest and smallest digit; Equation (9)), digit variance Var(d), the mean digit $\bar{d}=\frac{1}{D}\sum_{i=1}^{D}d_{i}$ (i.e., digit sum normalised by *D*; in (**C**), the Easy and Hard curves overlap), and the gap between the second and third largest digits $g_{2}$ for the two groups. Easy states consistently exhibit larger $g_{1}$ and, for some *D*, higher digit variance, whereas differences in digit sum are largely driven by the change in *D*.

**Figure 2:**
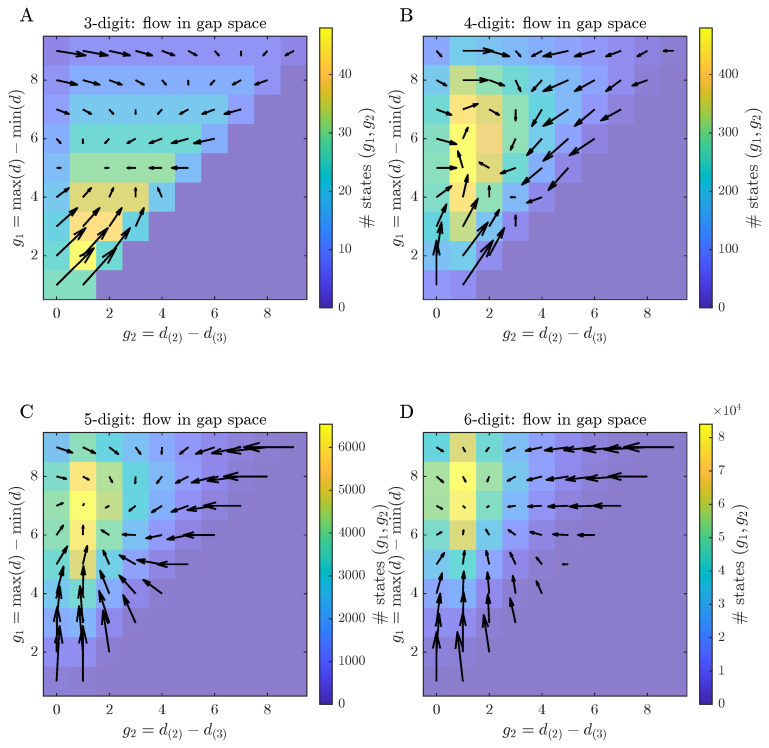
Flow fields in the gap space for D=3–6. Panels (**A**–**D**) correspond to D=3,4,5,6, respectively. Each panel shows the occupancy of gap states $\left(g_{1},g_{2}\right)$ under a uniform prior over ${𝒮}_{D}$ (colour scale) where # denotes the number (count) of underlying digit states in $S_{D}$ that fall into each gap cell. Arrows indicate the average one-step drift induced by a single Kaprekar step. Arrows indicate the mean change in gap coordinates per iteration, with horizontal and vertical components corresponding to $\Delta g_{2}$ and $\Delta g_{1}$, respectively. For each occupied gap state *g*, the arrow is the empirical mean drift $v_{D}\left(g\right)$ averaged over all underlying states $x\in {𝒮}_{D}\left(g\right)$ (Equation (13)). Within-cell variability around the mean drift is quantified by the increment covariance $\Sigma_{D}\left(g\right)$ (Equation (14)) and summarised in Appendix A.1.

**Figure 3:**
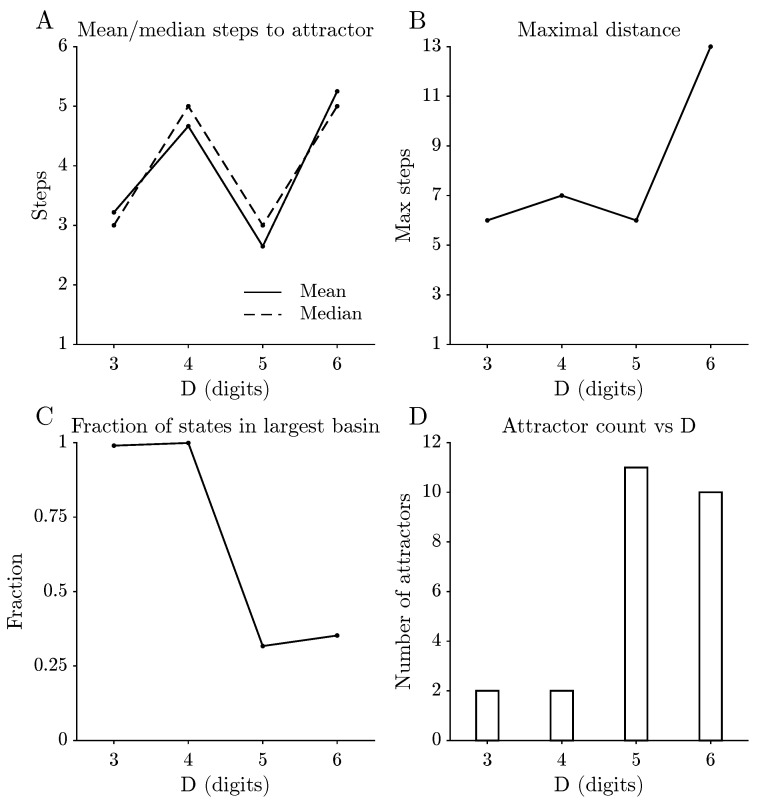
Global structure of Kaprekar dynamics as a function of digit length *D*. (**A**) Mean (solid) and median (dashed) number of iterations required to reach an attractor. (**B**) Maximal distance over all starting states. (**C**) Fraction of the state space contained in the largest basin of attraction. (**D**) Number of distinct attractors.

**Figure 4:**
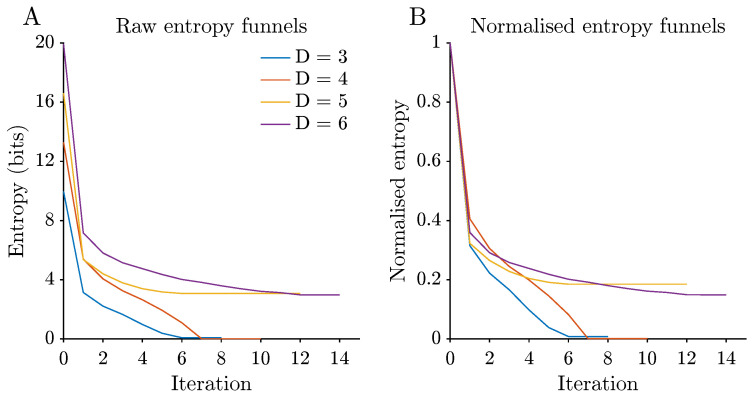
Entropy funnels across digit lengths. (**A**) Raw Shannon entropy of the induced distribution over attractors, conditional on trajectories that have converged by iteration *t*, for each *D*. (**B**) The same curves normalised as ${\tilde{H}}_{t}=\left(H_{t}-H_{\infty }\right)/\left(H_{0}-H_{\infty }\right)$, with $H_{\infty }$ set to the terminal value $H_{T}$ at the final plotted iteration *T* (chosen such that $T\geq t_{\max}$ for each *D*). All digit lengths exhibit a rapid early collapse of uncertainty, followed by a slower tail governed by the distribution of basin sizes.

**Figure 5:**
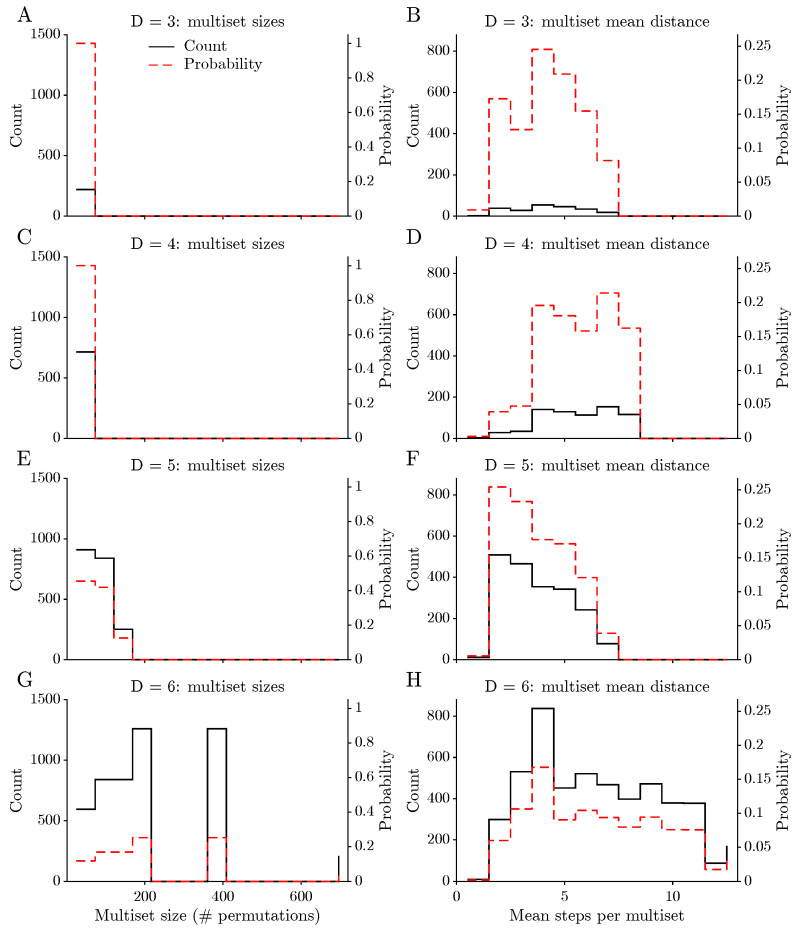
Multiset structure of Kaprekar dynamics across digit lengths. Panels (**A**,**C**,**E**,**G**) show the distributions of multiset sizes, i.e., the number of distinct permutations in each digit-multiset class, for D=3,4,5,6, respectively. Panels (**B**,**D**,**F**,**H**) show the corresponding distributions of mean distance to attractor per multiset class (average number of Kaprekar iterations taken by all states sharing the same multiset of digits). Solid black lines show absolute counts (left *y*-axis); dashed red lines show the corresponding normalised probabilities (right *y*-axis). As *D* increases, both the number and typical size of multiset classes grow sharply, and the distributions of multiset-averaged distances broaden and develop longer tails, indicating increasingly heterogeneous convergence behaviour. Note that most classes have a small mean distance (fewer than three steps), with a long right tail for D=5 and D=6.

**Figure 6:**
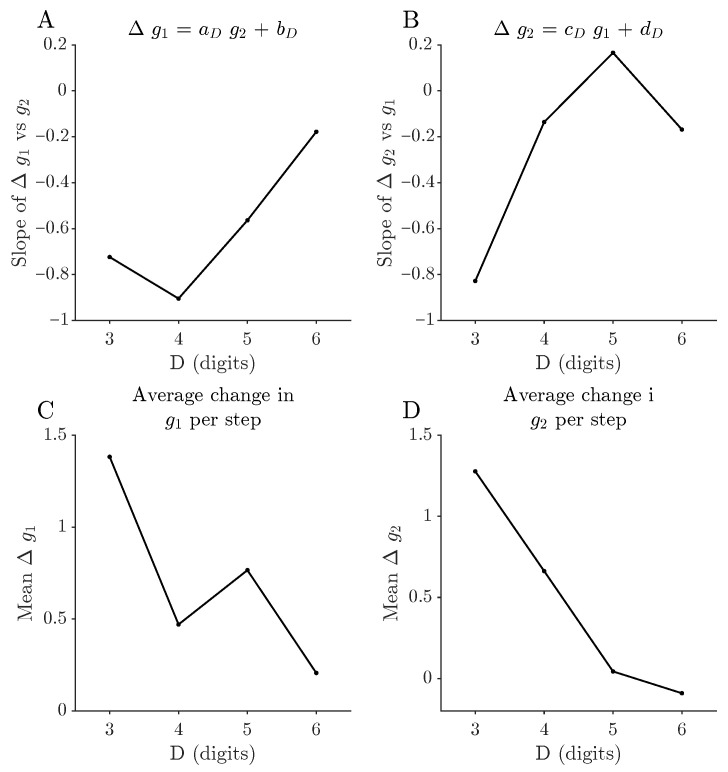
Cross-digit summary of average gap drift. Panels (**A**,**B**) show the slopes of the linear relations $\Delta g_{1}\approx a_{D}g_{2}+b_{D}$ and $\Delta g_{2}\approx c_{D}g_{1}+d_{D}$, respectively, as a function of *D*, obtained by ordinary least-squares fits across all occupied gap states. The negative slopes indicate a robust coupling between the two gap coordinates that weakens with increasing *D*. Panels (**C**,**D**) show the average drifts $\langle \Delta g_{1}\rangle$ and $\langle \Delta g_{2}\rangle$ per step, revealing a systematic tendency to increase $g_{1}$ and decrease $g_{2}$ that becomes smaller in magnitude for higher *D*.

**Figure 7:**
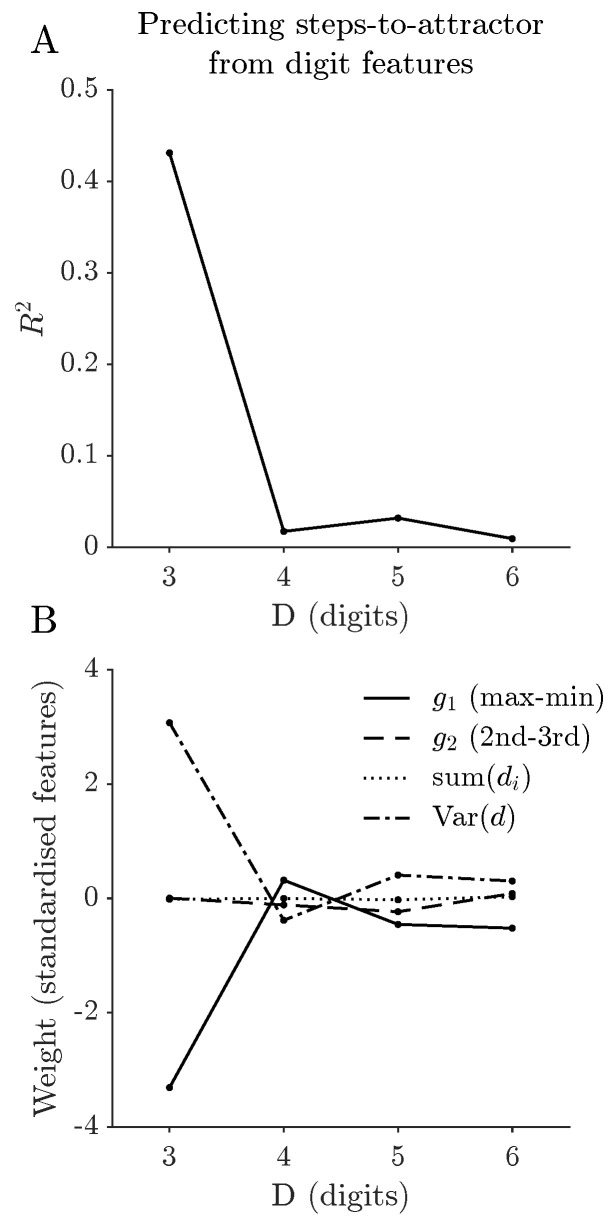
Predicting distance to attractor from simple digit features. Panel (**A**) shows the coefficient of determination $R^{2}$ for linear regression models predicting the number of Kaprekar steps from standardised features $\left(g_{1},g_{2},\mathrm{digit}\;\mathrm{sum},\mathrm{digit}\;\mathrm{variance}\right)$ as a function of digit length *D*. Predictive power is substantial for D=3 but drops sharply for D≥4 in this simple linear model. Panel (**B**) shows the learned regression weights for each feature across *D*, indicating that a large digit spread $g_{1}$ is strongly associated with faster convergence only for three digits, while for larger *D* the relationship between these local features and global distance to attractor becomes weak within this linear framework.

**Table 1:** Within-cell directional variability of the gap-space increment Δg across digit lengths *D*. For each occupied gap cell $g=(g_{1},g_{2})$, the circular dispersion $\sigma_{\theta }\left(g\right)$ is computed from increment directions Δg(x) over all states *x* mapping to that cell. Reported are the median and 90th percentile of $\sigma_{\theta }\left(g\right)$ over occupied cells, the fraction of cells with $\sigma_{\theta }\left(g\right)>1$ rad, and the number of occupied cells. # cells denotes the number of occupied gap cells (i.e., those with non-empty $S_{D}\left(g\right)$); for D∈{3,4,5,6} this count is 54.

*D*	Median $\sigma_{\theta }$	$p_{90}\left(\sigma_{\theta }\right)$	$\mathrm{Pr}[\sigma_{\theta }>1]$	# Cells
3	0	$1.55\times 10^{-8}$	0	54
4	0	$5.03\times 10^{-8}$	0	54
5	0.173	0.903	0.0741	54
6	0.284	1.180	0.185	54

**Table 2:** Summary of attractor cycles for D=5,6 and their basin weight distribution under the uniform initial ensemble on ${𝒮}_{D}$. $K_{D}$ denotes the number of distinct attractor cycles; $w_{i}$ are the largest basin weights in descending order; and $H_{\infty }$ is the corresponding basin size entropy in bits.

*D*	$K_{D}$	$w_{1}$	$w_{2}$	$w_{3}$	$H_{\infty }$
5	3	0.484848	0.483248	0.031903	1.171943
6	3	0.935529	0.062521	0.001950	0.357554

**Figure A1:**
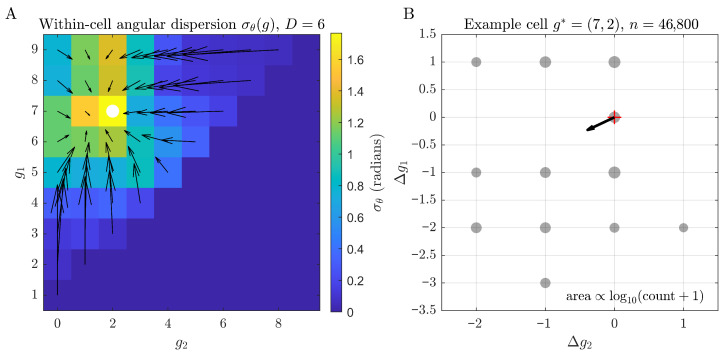
Within-cell variability of the gap-space drift for D=6. (**A**) Angular dispersion $\sigma_{\theta }\left(g\right)$ of increment directions Δg(x) within each occupied gap cell $g=(g_{1},g_{2})$, with mean drift arrows (conditional means $v_{D}\left(g\right)$) overlaid; the marked cell indicates the example $g^{*}$. (**B**) Discrete one-step increments $\left(\Delta g_{2},\Delta g_{1}\right)$ observed from the example cell $g^{*}$, with the marker area proportional to $\log_{10}\left(\mathrm{count}+1\right)$; the arrow shows the mean increment for that cell.

**Figure A2:**
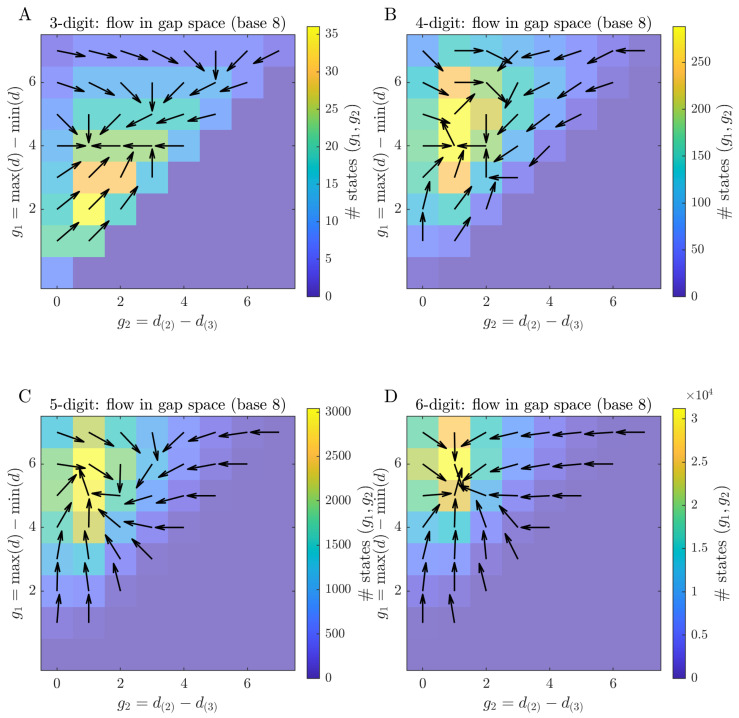
Flow fields in the gap space for base 8 and D=3–6 using the main-text definition $g_{2}=d_{\left(2\right)}-d_{\left(3\right)}$ (*mid*). Panels (**A**–**D**) correspond to D=3,4,5,6, respectively. Each panel shows the occupancy of gap states $\left(g_{1},g_{2}\right)$ under a uniform prior over ${𝒮}_{D}$ (colour scale), where # denotes the number (count) of underlying base-8 digit states in each gap cell. Arrows indicate the average one-step drift induced by a single Kaprekar step; horizontal and vertical components correspond to $\Delta g_{2}$ and $\Delta g_{1}$, respectively.

**Figure A3:**
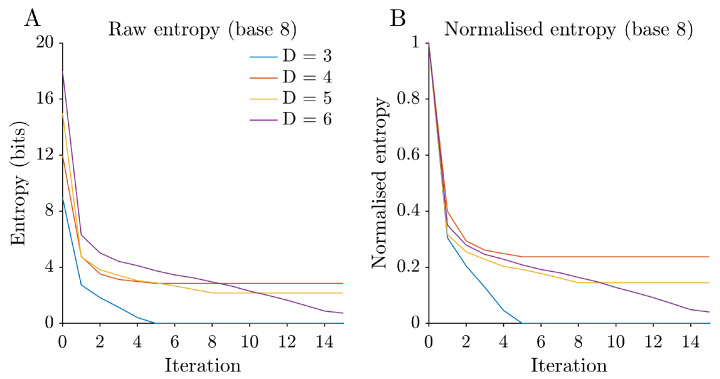
Entropy funnels in base 8 (mid definition). (**A**) Raw Shannon entropy of the induced distribution over attractors, conditional on trajectories that have converged by iteration *t*, for each *D* (base 8; D∈{3,4,5,6}). (**B**) The same curves normalised as ${\tilde{H}}_{t}=\left(H_{t}-H_{\infty }\right)/\left(H_{0}-H_{\infty }\right)$, with $H_{\infty }$ set to the terminal value $H_{T}$ at the final plotted iteration *T* (chosen such that $T\geq t_{\max}$ for each *D*). Analog of Figure 4 computed for base 8 using the same entropy-funnel procedure as in the main text, under the *mid* definition $g_{2}=d_{\left(2\right)}-d_{\left(3\right)}$.

**Figure A4:**
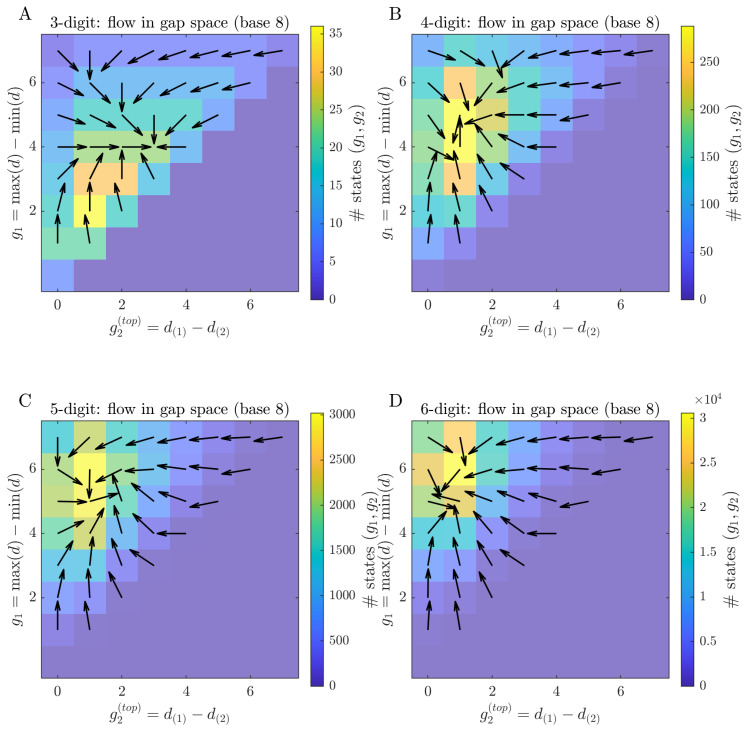
Flow fields in the gap space for base 8 and D=3–6 using the *top* definition $g_{2}^{\left(\mathrm{top}\right)}=d_{\left(1\right)}-d_{\left(2\right)}$. Panels (**A**–**D**) correspond to D=3,4,5,6, respectively. Each panel shows the occupancy of gap states $\left(g_{1},g_{2}\right)$ under a uniform prior over ${𝒮}_{D}$ (colour scale), where # denotes the number (count) of underlying base-8 digit states in each gap cell. Arrows indicate the average one-step drift induced by a single Kaprekar step; horizontal and vertical components correspond to $\Delta g_{2}$ and $\Delta g_{1}$, respectively. Same plotting conventions as Figure A2.

**Figure A5:**
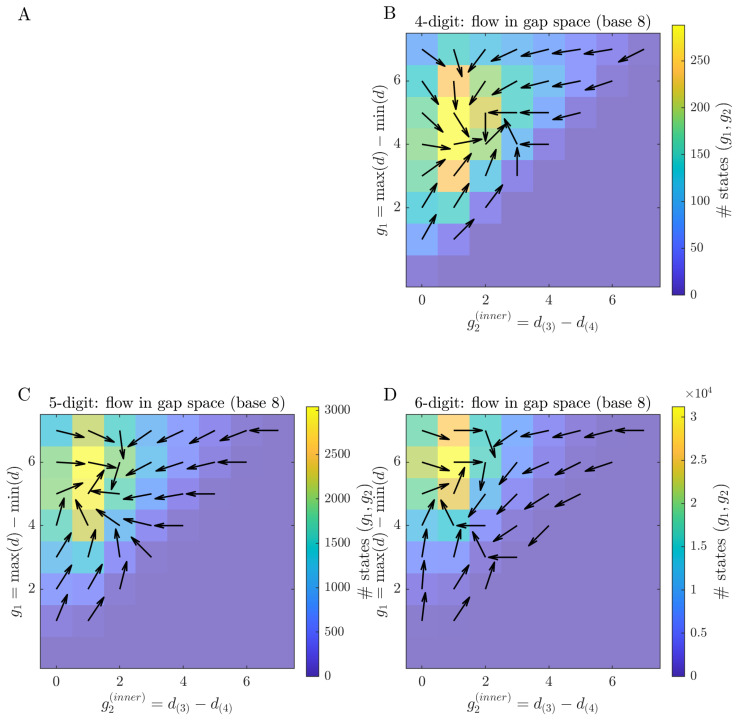
(**A**) Flow fields in the gap space for base 8 using the *inner* definition $g_{2}^{\left(\mathrm{inner}\right)}=d_{\left(3\right)}-d_{\left(4\right)}$, which is defined only for D≥4 (hence no panel for D=3). Panels (**B**–**D**) correspond to D=4,5,6, respectively. Each panel shows the occupancy of gap states $\left(g_{1},g_{2}\right)$ under a uniform prior over ${𝒮}_{D}$ (colour scale), where # denotes the number (count) of underlying base-8 digit states in each gap cell. Arrows indicate the average one-step drift induced by a single Kaprekar step; horizontal and vertical components correspond to $\Delta g_{2}$ and $\Delta g_{1}$, respectively. Same plotting conventions as Figure A2.

## Data Availability

All data analysed in this study consist of deterministic outputs of the Kaprekar map in base 10 for digit lengths D=3–6. The MATLAB code used to enumerate the state spaces, compute all summary statistics, and generate the figures is openly available at https://github.com/ChristophDahl/kaprekar-routine-analysis (accessed on 22 November 2025) under the MIT licence. The repository includes a master script (run_kaprekar_all.m) that reproduces all analyses and figures reported in this manuscript.

## Appendix A. Supplementary Analyses

### Appendix A.1. Within-Cell Drift Variability

In the main text, arrows in Figure 2 depict the conditional mean gap increment $v_{D}\left(g\right)=E\left[\Delta g\left(x\right)|g\left(x\right)=g\right]$, where $\Delta g\left(x\right)=g(K_{D}\left(x\right))-g\left(x\right)$ and the average is taken over all underlying states $x\in S_{D}\left(g\right)=\left\{x\in S_{D}\;:\;g\left(x\right)=g\right\}$. Because many distinct digit configurations can map to the same gap cell *g*, it is useful to quantify how much the one-step increment Δg(x) varies within a cell.

For each occupied gap state *g*, the empirical covariance of increments is computed as in Equation (14), and dispersion is summarised using (i) total increment variance $\mathrm{tr}(\Sigma_{D}\left(g\right))$ and (ii) an angular dispersion measure $\sigma_{\theta }\left(g\right)$ computed from the directions of Δg(x). Figure A1 visualises these quantities and provides a representative example cell showing the distribution of increments around the mean drift arrow. In the Results, Table 1 summarises these within-cell dispersion statistics across digit lengths D∈{3,4,5,6}, showing that directional variability is negligible for D=3,4 but becomes non-negligible for D≥5. A concrete visualisation is provided for the representative case D=6. In the right panel, marker area is proportional to $\log_{10}\left(\mathrm{count}+1\right)$, where count is the number of underlying states $x\in S_{D}\left(g^{*}\right)$ that realise a given discrete increment $\left(\Delta g_{2},\Delta g_{1}\right)$.

### Appendix A.2. Robustness to Alternative Gap-Pair Definitions

To assess whether the qualitative gap-space flow patterns depend strongly on the specific choice of the internal gap $g_{2}$, occupancy and mean drift fields were recomputed under two alternatives to the main-text definition $g_{2}=d_{\left(2\right)}-d_{\left(3\right)}$: $g_{2}^{\left(\mathrm{top}\right)}=d_{\left(1\right)}-d_{\left(2\right)}$ and (for D≥4) $g_{2}^{\left(\mathrm{inner}\right)}=d_{\left(3\right)}-d_{\left(4\right)}$. For each definition, drift fields were constructed by averaging $\left(\Delta g_{2},\Delta g_{1}\right)$ over all states consistent with a given gap cell, analogously to Equation (13). Figure A2–Figure A5 show gap-space flows for base 8 and D∈{3,4,5,6} under the main-text (*mid*) definition and two alternatives (*top* and *inner*). Figure A3 shows entropy funnels for base 8 computed under the same *mid* definition. Table A1 reports summary similarity statistics (occupancy correlation and drift vector cosine similarity) relative to the main-text *mid* definition.

**Table A1:** Similarity of occupancy and mean drift fields for alternative $g_{2}$ definitions, relative to the main-text definition $g_{2}=d_{\left(2\right)}-d_{\left(3\right)}$ (mid). Occupancy similarity is measured as the Pearson correlation of gap-state occupancies across occupied cells; drift similarity is measured as the mean cosine similarity between drift vectors over cells occupied under both definitions.

*D*	corr(*N*) (Top)	corr(*N*) (Inner)	〈cos〉 (Top)	〈cos〉 (Inner)
3	1.000	–	0.595	–
4	0.998	–	0.612	–
5	0.987	0.972	0.641	0.618
6	0.981	0.963	0.658	0.631

Qualitatively, the geometry of the gap-space flow is preserved across these alternative $g_{2}$ definitions. Table A1 summarises occupancy and drift field similarity relative to the main-text *mid* definition.

### Appendix A.3. Attractors and Basin Sizes

For completeness, Appendix A.3 reports the attractor cycles for D=5 and D=6 together with their basin sizes under the Kaprekar map. For an attractor cycle *C*, the basin size is $\left|B\left(C\right)|=|\{x\in {𝒮}_{D}:\alpha \left(x\right)=C\}\right|$, and the corresponding basin weight under the uniform initial ensemble on ${𝒮}_{D}$ is $p_{\infty }\left(C\right)=\left|B\left(C\right)|/\right|{𝒮}_{D}|$. Cycle length is l=|C|, with fixed points corresponding to the special case l=1.

As an illustration (for D=6), the initial state x=873,210 maps to 860,832 in one step (cycle entry at t=1), while x=990,010 maps to ⋯ and first enters the same attractor at t=8. After entry, the trajectory follows the 7-cycle 860,832→862,632→642,654→420,876→851,742→750,843→840,852→860,832. All observed attractors for D∈{5,6} are periodic orbits of the deterministic map $K_{D}$ (fixed points are the special case l=1). No non-periodic attractors occur on a finite state space.

**Table A2:** Attractor cycles for D=5 (base 10) and their basin sizes.

Cycle (Canonical)	*ℓ*	Basin Size	Basin Weight $p_{\infty }$
53,955 → 59,994	2	3190	0.0319031903
61,974 → 82,962 → 75,933 → 63,954	4	48,480	0.4848484848
62,964 → 71,973 → 83,952 → 74,943	4	48,320	0.4832483248

**Table A3:** Attractor cycles for *D* = 6 (base 10) and their basin sizes.

Cycle (Canonical)	*ℓ*	Basin Size	Basin Weight $p_{\infty }$
420,876 → 851,742 → 750,843 → 840,852 → 860,832 → 862,632 → 642,654	7	935,520	0.9355293553
631,764	1	62,520	0.0625206252
549,945	1	1950	0.0019500195

### Appendix A.4. Linear Regression Details and Diagnostics

**Table A4:** Multiple linear regression predicting steps to attractor from *z*-scored digit features. Coefficients are reported with standard errors (SEs), 95% confidence intervals (CIs), and two-sided *p*-values. Fit statistics (*N*, $R^{2}$, adj. $R^{2}$, and RMSE) are reported per *D*.

*D*	Predictor	Estimate	SE	CI_low_	CI_high_	*p*
D=3: N=998, $R^{2}=0.431$, *adj*. $R^{2}=0.429$, *RMSE* = 1.077						
3	Intercept	3.2244	0.0342	3.1574	3.2915	0
3	$g_{1}$	−3.3113	0.1239	−3.5545	−3.0681	6.03 × 10^−119^
3	$g_{2}$	0.0042	0.0439	−0.0820	0.0904	0.924
3	sum($d_{i}$)	−0.0149	0.0362	−0.0859	0.0561	0.680
3	Var(*d*)	3.0743	0.1213	2.8363	3.3123	1.04 × 10^−109^
D=4: N=9998, $R^{2}=0.017$, *adj*. $R^{2}=0.017$, *RMSE* = 1.763						
4	Intercept	4.6655	0.0176	4.6310	4.7001	0
4	$g_{1}$	0.3182	0.0591	0.2024	0.4340	7.31 × 10^−8^
4	$g_{2}$	−0.1144	0.0250	−0.1635	−0.0654	4.94 × 10^−6^
4	sum($d_{i}$)	−0.0011	0.0176	−0.0357	0.0335	0.950
4	Var(*d*)	−0.3832	0.0663	−0.5132	−0.2532	7.70 × 10^−9^
D=5: N=50,000, $R^{2}=0.029$, *adj*. $R^{2}=0.029$, *RMSE* = 1.438						
5	Intercept	2.6461	0.0064	2.6335	2.6587	0
5	$g_{1}$	−0.4562	0.0160	−0.4875	−0.4249	8.10 × 10^−178^
5	$g_{2}$	−0.2163	0.0077	−0.2313	−0.2013	9.49 × 10^−174^
5	sum($d_{i}$)	−0.0242	0.0065	−0.0369	−0.0114	2.03 × 10^−4^
5	Var(*d*)	0.4052	0.0172	0.3715	0.4388	1.11 × 10^−122^
D=6: N=50,000, $R^{2}=0.010$, *adj*. $R^{2}=0.010$, *RMSE* = 3.105						
6	Intercept	5.2345	0.0139	5.2073	5.2618	0
6	$g_{1}$	−0.5602	0.0287	−0.6166	−0.5039	2.85 × 10^−84^
6	$g_{2}$	0.0770	0.0152	0.0473	0.1067	3.83e × 10^−7^
6	sum($d_{i}$)	0.0128	0.0142	−0.0150	0.0407	0.367
6	Var(*d*)	0.3450	0.0296	0.2869	0.4031	2.67 × 10^−31^
